# Evolution of carbapenemase activity in the class C β-lactamase ADC-1

**DOI:** 10.1128/mbio.00185-25

**Published:** 2025-04-28

**Authors:** Nichole K. Stewart, Marta Toth, Monolekha Bhattacharya, Clyde A. Smith, Sergei B. Vakulenko

**Affiliations:** 1 Department of Chemistry and Biochemistry, University of Notre Dame, Notre Dame, Indiana, USA; 2 Stanford Synchrotron Radiation Lightsource, Stanford University, Menlo Park, California, USA; 3 Department of Chemistry, Stanford University, Stanford, California, USA; Louis Stokes Veterans Affairs Medical Center, Cleveland, Ohio, USA

**Keywords:** class C carbapenemase, carbapenem, antibiotic resistance, crystal structure, kinetics, *Acinetobacter baumannii*

## Abstract

Antibiotic resistance in bacteria poses a significant threat to public health. Among dozens of available antimicrobial agents, carbapenems are used as drugs of choice for the treatment of serious infections caused by pathogens resistant to other antibiotics. However, their usefulness has been severely compromised due to the emergence and wide spread of carbapenem-resistant clinical isolates worldwide. High-level resistance to carbapenems in bacteria is mediated by the production of β-lactamases from three molecular classes, A, B, and D, but not by class C enzymes. In this study, we selected a triple mutant of the intrinsic class C *Acinetobacter*-derived cephalosporinase ADC-1 (ADC-1_TM_) that confers high-level resistance to the carbapenems meropenem, ertapenem, and doripenem. Kinetic experiments demonstrated that the apparent binding affinity, along with the acylation and deacylation rates, were all improved for the mutant enzyme. X-ray crystallography, molecular docking, and molecular dynamics simulations revealed that the amino acid substitutions in ADC-1_TM_ produce significant changes in the enzyme active site architecture and binding mode of the carbapenem ertapenem. These changes allow for better positioning of a deacylating water for nucleophilic attack, thus explaining the significantly improved rate of ertapenem deacylation by ADC-1_TM_. In this study, we showed for the first time that a class C β-lactamase can produce high-level resistance to carbapenem antibiotics, which underlines the potential for enzymes of this class to evolve such resistance and could further exacerbate the problem of antibiotic resistance in bacteria.

**IMPORTANCE:**

Carbapenems belong to the most widely used family of β-lactam antibiotics and are considered drugs of choice for difficult-to-treat and often deadly infections. Widespread carbapenem-resistant isolates have drastically diminished the utility of these important antibiotics and resulted in high mortality rates. Resistance to carbapenems in clinical pathogens is mainly due to the production of β-lactamases, enzymes that destroy these drugs. Out of the four molecular classes of β-lactamases, various enzymes belonging to classes A, B, and D produce high levels of resistance to carbapenems; however, enzymes of class C have failed to evolve such resistance. Here, we demonstrate that the intrinsic ADC-1 β-lactamase of the clinically important pathogen *Acinetobacter baumannii* can evolve high-level resistance to carbapenems by just three amino acid substitutions and disclose the molecular mechanisms of its carbapenemase activity. This study demonstrates the potential for the evolution of carbapenemase activity in class C β-lactamases.

## INTRODUCTION

The discovery of antibiotics and their introduction into clinical practice revolutionized the treatment of infectious diseases and has saved millions of lives. Currently, more than 100 antibiotics belonging to a dozen classes are available; however, bacteria are continually evolving resistance to all newly introduced antibacterials (1). As a result, multidrug-resistant pathogens are currently circulating worldwide, severely compromising available therapeutic options. The carbapenems from the β-lactam family of antibiotics are often used as drugs of last resort for the treatment of infections caused by such bacteria (2). Alarmingly, resistance to these drugs in gram-negative bacteria has also emerged, thus further limiting effective treatments and causing an increase in the severity of infections and mortality rates.

Several mechanisms can contribute to carbapenem resistance, including alteration of the antibiotic target, the penicillin-binding proteins or PBPs, increased drug efflux out of the cell, and decreased drug penetration due to modification or loss of membrane porins (3). By far, the most common mechanism is the production of carbapenemases, enzymes of the β-lactamase family that inactivate the carbapenems (4). Among the four molecular classes of β-lactamases, many enzymes of classes A, B, and D produce high levels of resistance to carbapenems in various bacterial species of gram-negative bacteria. For the class C β-lactamases, kinetic studies with the carbapenem imipenem showed that some of them, such as CMY-2, CMY-10, ACT-1, and ACT-28, possess only very weak carbapenemase activity (5–7). These enzymes produced clinical levels of resistance to carbapenems only in strains with diminished outer membrane permeability resulting from the loss or mutation of porins (6, 8–10).

One of the organisms that was impacted by the wide spread of carbapenemases is *Acinetobacter baumannii*. Carbapenem-resistant *A. baumannii* (CR*Ab*) has been categorized by both the Centers for Disease Control and the World Health Organization as the highest public health threat, which requires immediate intervention (11, 12). This bacterium is a major nosocomial pathogen that frequently causes ventilator-associated pneumonia, bacteremia, endocarditis, and wound and urinary tract infections (13). Frequently, CR*Ab* isolates are also resistant to most other available drugs, occasionally increasing mortality rates up to 85% (14–18). Moreover, during the COVID-19 pandemic, there was an alarming increase (78%) in the incidence of nosocomial CR*Ab* infections (19). The class D enzymes play the leading role in conferring resistance to carbapenems in *A. baumannii*, while class B zinc metalloenzymes and class A carbapenemases are found less frequently (20).

In 2014, it was reported that the β-lactamase ADC-68 confers resistance to carbapenem antibiotics in a clinical isolate of *A. baumannii* (21). ADC-68 is a representative of a group of intrinsic chromosomally encoded class C *Acinetobacter*-derived cephalosporinases (ADCs), with more than 350 variants currently reported (10, 22). These enzymes can produce resistance to penicillins, narrow- and expanded-spectrum cephalosporins, and monobactams (10). ADC-68 differs from ADC-1, which is not a carbapenemase, by seven amino acid substitutions and is still the only class C enzyme reported to confer resistance to carbapenems by itself (21). However, the role of these substitutions in carbapenemase activity was not evaluated. To study the evolutionary pathways of ADC enzymes and elucidate the number of substitutions required for carbapenemase activity, we performed a selection for carbapenem-resistant *A. baumannii* expressing ADC-1 in the presence of carbapenem antibiotics. We succeeded in obtaining such a mutant and show that only three amino acid substitutions are sufficient to convert the enzyme into a carbapenemase producing high levels of resistance to carbapenems. Here, we present studies of the mechanism of resistance to carbapenems of this ADC-1 triple mutant enzyme (hereinafter referred to as ADC-1_TM_).

## RESULTS AND DISCUSSION

### Selection of ADC-1 mutants and effect on resistance to β-lactams and bacterial growth

Since *A. baumannii* strains encode the intrinsic class D β-lactamase OXA-51, which can gain high-level carbapenemase activity as a result of a single mutation (23, 24), the selection of carbapenem-resistant mutants of ADC-1 was performed in *Escherichia coli* DH5α expressing ADC-1 from the pHF016 vector. Selection in media containing imipenem was unsuccessful. However, successive serial passages in media with increasing concentrations of ertapenem yielded the ADC-1_TM_ mutant containing the Val292Phe, Ser318Thr, and Phe322Ser substitutions (SANC residue numbering [25]). The MIC of ertapenem against this strain (1 µg/mL) was 64-fold higher than the MIC produced by the parental enzyme. As ADC-1 is an intrinsic β-lactamase of *A. baumannii*, the *bla*_ADC-1TM_ gene in a shuttle vector was introduced into *A. baumannii* ATCC 17978 for further studies. To evaluate the contribution of each of the three ADC-1 substitutions to carbapenem resistance, we also introduced each of them individually and in combination. The strain expressing ADC-1_TM_ produced high levels of resistance, with MICs increased from 0.25 to 16 µg/mL for meropenem and doripenem and from 4 to 128 µg/mL for ertapenem compared to the *A. baumannii* strain expressing ADC-1 (Table 1). No change in the MIC of imipenem was detected. For meropenem and doripenem, each amino acid substitution contributed to resistance, while for ertapenem, the Val292Phe substitution had the largest impact on MICs. These results show that the three substitutions in ADC-1_TM_ increase MICs of meropenem, ertapenem, and doripenem in an additive manner to result in high-level carbapenem resistance. Search for the three substitutions in more than 350 reported ADC β-lactamase sequences revealed the presence of Ser318Thr in three clinical isolates (ADC-145, ADC-200, and ADC-256) (22). Whether the selection of mutants like ADC-1_TM_, which require three amino acid substitutions, would occur in the clinic remains to be seen. However, the presence of the intrinsic class D β-lactamase OXA-51 in *A. baumannii*, which can produce a high level of resistance to carbapenems as a result of just one mutation (23, 24), would reduce the selective pressure and may hinder the evolution of carbapenemase activity in ADC-1.

**TABLE 1:** MICs (µg/mL) of β-lactams against *A. baumannii* ATCC 17978 producing ADC-1, ADC-1_TM_, and ADC-68

Enzyme	MIC (µg/mL)				
	MEM	ETP	DOR	IPM	AMP
ADC-1	0.25	4	0.25	0.5	2,048
Val292Phe ADC-1	1	32	1	0.5	512
Ser318Thr ADC-1	0.5	8	1	0.5	512
Phe322Ser ADC-1	0.5	8	0.5	0.5	1,024
Val292Phe/Ser318Thr ADC-1	2	32	4	0.5	64
Val292Phe/Phe322Ser ADC-1	2	32	4	0.5	512
Ser318Thr/Phe322Ser ADC-1	2	32	4	1	1,024
ADC-1_TM_	16	128	16	0.5	256
ADC-68	0.25 (4*^a^*)	4 (16)	0.25 (ND*^b^*)	0.5 (8)	4,096 (256)
Control*^c^*	0.25	4	0.25	0.25	64

^*a*^ Values in parentheses are from reference 21.

^*b*^ ND, not determined.

^*c*^ Parental *A. baumannii* ATCC 17978 strain not expressing any β-lactamase from a plasmid.

We also monitored the growth rates of *A. baumannii* expressing ADC-1 or ADC-1_TM_ and observed that they were very similar (Fig. S1). These data show that the three mutational changes leading to carbapenemase resistance in ADC-1_TM_ do not significantly affect bacterial growth.

Previously, it was reported that the ADC-68 β-lactamase produces resistance to carbapenems (21). The mature enzyme has seven amino acid substitutions compared to ADC-1, all of which are different from those identified in ADC-1_TM_. To compare the level of resistance produced by ADC-1_TM_ with that of ADC-68, the gene encoding ADC-68 was cloned under the same promoter into our shuttle vector and expressed in the *A. baumannii* ATCC 17978 strain. Surprisingly, we did not observe any increase in the MICs of the four carbapenems tested (Table 1). However, ADC-68 produced a 16-fold higher MIC of ampicillin than that reported (21) and a twofold higher MIC than that of ADC-1, indicating that the enzyme was active and expressed from our vector at a high level. Combined, these data demonstrate that ADC-1_TM_ produces high levels of resistance to meropenem, ertapenem, and doripenem, while under the same conditions, no carbapenem resistance was detected for ADC-68.

### Enzyme kinetics

Next, we evaluated the steady-state kinetic parameters *k*_cat_ and *K*_*m*_ for both ADC-1 and ADC-1_TM_. The former is a rate constant that describes the maximum rate of turnover or the rate-limiting step, while the latter is a measure of the apparent affinity of an enzyme for a substrate. For meropenem, ertapenem, and doripenem, the *k*_cat_ values of ADC-1_TM_ were increased 41-, 86-, and 18-fold, respectively, relative to ADC-1, while the *K*_*m*_ values were nearly four- and sixfold lower for meropenem and doripenem, respectively, and below the detection limit (≤1 µM) for ertapenem (Table 2). These results indicate that the amino acid substitutions in ADC-1_TM_ both increase the rate of turnover and lead to a stronger apparent binding affinity for these carbapenems relative to ADC-1, though the effect is greater on the former. The combination of changes in the *k*_cat_ and *K*_*m*_ values for ADC-1_TM_ with meropenem and doripenem resulted in significant (158- and ≥110-fold, respectively) increases in the catalytic efficiencies (*k*_cat_/*K*_*m*_) relative to ADC-1, while for ertapenem, based on the upper limits of *K*_*m*_, it was increased 86-fold (Table 2). For imipenem, there was very little change in the *k*_cat_ value, while only the upper limit of the *K*_*m*_ value (≤1 µM) could be measured. These data show that ADC-1_TM_ has higher catalytic efficiency than ADC-1 for meropenem, ertapenem, and doripenem, which is reflected in the clinically relevant levels of resistance produced by the enzyme for these carbapenems (Table 1).

**TABLE 2:** Steady-state kinetic parameters of ADCs

Antibiotic	Enzyme	*k*_cat_ (s^−1^)	*K*_*m*_ (µM)	*k*_cat_/*K*_*m*_ (M^−1^ s^−1^)
MEM	ADC-1	0.0034 ± 0.0001	11 ± 1	(3.1 ± 0.3) × 10^2^
	ADC-1_TM_	0.14 ± 0.01	2.9 ± 0.3	(4.9 ± 0.4) × 10^4^
	ADC-68	0.028 ± 0.001	50 ± 4	(5.5 ± 0.4) × 10^2^
ETP	ADC-1	0.00037 ± 0.00002	≤1	≥3.7 × 10^2^
	ADC-1_TM_	0.032 ± 0.001	≤1	≥3.2 × 10^4^
	ADC-68	0.011 ± 0.001	3.5 ± 0.7	(3.3 ± 0.7) × 10^3^
DOR	ADC-1	0.0018 ± 0.0001	6.0 ± 0.4	(3.0 ± 0.2) × 10^2^
	ADC-1_TM_	0.033 ± 0.001	≤1	≥3.3 × 10^4^
	ADC-68	0.0049 ± 0.0001	6.0 ± 0.4	(8.2 ± 0.6) × 10^2^
IPM	ADC-1	0.0010 ± 0.0001	1.3 ± 0.2	(8.1 ± 1.1) × 10^2^
	ADC-1_TM_	0.00060 ± 0.00002	≤1	≥6.0 × 10^2^
	ADC-68	0.0036 ± 0.0001	1.0 ± 0.2	(3.6 ± 0.7) × 10^3^
AMP	ADC-1	4.8 ± 0.1	8.6 ± 0.8	(5.5 ± 0.5) × 10^5^
	ADC-1_TM_	0.74 ± 0.03	13 ± 2	(5.7 ± 0.9) × 10^4^
	ADC-68	3.2 ± 0.1	≤5	≥6.5 × 10^5^

To gain deeper insights into the kinetics of ADC-1_TM_, we evaluated the rate constants for acylation (*k*_2_) and deacylation (*k*_3_), which allows for discernment of the rate-limiting step of the catalytic reaction. We chose ertapenem for these experiments, as the highest increase in the *k*_cat_ value of ADC-1_TM_ was observed with this carbapenem. Under single-turnover conditions, acylation of ADC-1 and ADC-1_TM_ by ertapenem was biphasic, with half of the reaction completed within seconds, and the remaining finished in minutes for both enzymes. This behavior was previously reported for some class A and D enzymes (26–31), but not for class C β-lactamases. For both enzymes, the acylation rate of the fast phase (*k*_2 fast_) increased with increasing concentration of enzyme, and saturation was not reached. The *k*_2 fast_ value was at least 62-fold higher for ADC-1_TM_ than for ADC-1 (*k*_2 fast_ = >68 ± 5 s^−1^ and 1.1 ± 0.1 s^−1^, respectively). In contrast, the acylation rate of the slow phase of the reaction (*k*_2 slow_) for both enzymes did not increase with increasing enzyme concentration, and the *k*_2 slow_ value was 3.6-fold higher for ADC-1_TM_ (*k*_2 slow_ = 0.043 ± 0.001 s^−1^) than for ADC-1 (*k*_2 slow_ = 0.012 ± 0.001 s^−1^). These results show that both phases of acylation are improved in ADC-1_TM_ compared to ADC-1, though the effect is greater on *k*_2 fast_.

Next, we attempted to evaluate the deacylation rate constant, *k*_3_, using the jump dilution method. Upon the 100-fold recommended dilution (32) of either ADC-1 or ADC-1_TM_ into the control reaction (without ertapenem), we observed that the rate was lower than expected and also was not linear, which prevented the determination of this rate constant by this method. Therefore, for ADC-1, we used the relationship *k*_3_ = (*k*_cat_ × *k*_2_)/(*k*_2_ − *k*_cat_) to calculate *k*_3_. Using either *k*_2_ value (*k*_2 fast_ or *k*_2 slow_) for the calculation gave the same *k*_3_ value of 0.00037 s^−1^, which is identical to *k*_cat_ (Table 2) and thus is the rate-limiting step for this enzyme. However, for ADC-1_TM_, the calculated *k*_3_ values using *k*_2 fast_ or *k*_2 slow_ were different (0.032 and 0.13 s^−1^, respectively). This does not allow for unequivocal determination of the rate-limiting step of the reaction, since the former value, which is identical to *k*_cat_, would indicate that deacylation is rate limiting for ADC-1_TM_, while the latter value, which is faster than *k*_cat_, shows that the slow phase of acylation is rate limiting. To clarify this, we tried once more to measure the *k*_3_ value experimentally by using different enzyme dilutions. With a 20-fold dilution, the issues with the control were resolved, and the *k*_3_ value determined under this condition was 0.063 ± 0.001 s^−1^. This value, which is likely underestimated as it was obtained using a fivefold smaller than recommended dilution, is still larger than the *k*_cat_ value (Table 2), indicating that unlike for ADC-1, where deacylation is rate limiting for turnover of ertapenem, for ADC-1_TM_, the slow phase of acylation is the rate-limiting step.

We also evaluated the steady-state kinetics for ADC-68. For ampicillin, there was only a small decrease (1.5-fold) in *k*_cat_ compared to ADC-1, while only the upper limit could be determined for the *K*_*m*_ value (≤5 µM), which gave a higher catalytic efficiency than that of the parental enzyme (Table 2). This result shows that ADC-68 is at least as active as ADC-1 and correlates well with the MIC result for ampicillin obtained in this study (Table 1). The catalytic efficiency of ADC-68 for ampicillin is also at least 1.9-fold higher than that reported, which demonstrates that the enzyme is more active than that described in the literature (21). The *k*_cat_ values for meropenem, doripenem, and imipenem increased approximately three- to eightfold compared to those of ADC-1, and the *K*_*m*_ values were either nearly the same or increased 4.5-fold (Table 2). Consequently, the catalytic efficiencies of ADC-68 were increased only marginally (approximately two- to fourfold) relative to ADC-1. For ertapenem, we cannot unequivocally determine which enzyme is more active, as only the upper limit (≤1 µM) could be determined for the *K*_*m*_ value of ADC-1, allowing for the determination of only the lower limit of its catalytic efficiency.

Compared to ADC-1_TM_, the *k*_cat_ values of ADC-68 were 5-, 2.9-, and 6.7-fold lower for meropenem, ertapenem, and doripenem, respectively, while the *K*_*m*_ values were increased 17-, ≥3.5-, and ≥6-fold (Table 2), indicating that the rate of turnover by ADC-68 is slower, and the relative binding affinity is weaker than that of ADC-1_TM_. As a result, its catalytic efficiencies for these three carbapenems were 89-, ≥9.7-, and ≥40-fold lower, respectively. In contrast, for imipenem, the *k*_cat_ value of ADC-68 was sixfold higher than that of our ADC-1 triple mutant. As only the upper limit (≤1 µM) could be determined for the *K*_*m*_ value for ADC-1_TM_, its catalytic efficiency for imipenem cannot be determined unequivocally, precluding comparison with that of ADC-68. These results show that while the carbapenemase activity of ADC-68 is slightly higher than that of ADC-1, it is much lower than that of ADC-1_TM_. Our data are in disagreement with those earlier published (21), as we show that ADC-68 lacks potent carbapenemase activity and is not capable of producing clinical levels of resistance in *A. baumannii*.

### Structural studies

**Fig 1:**
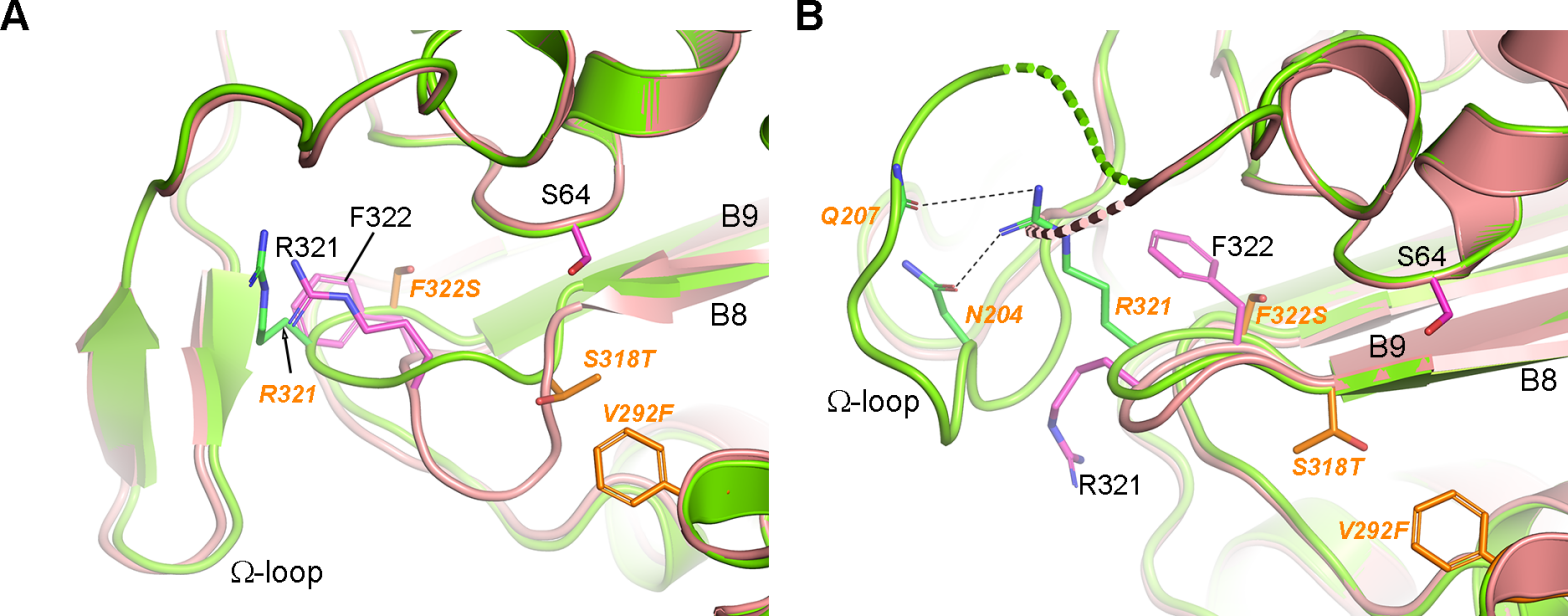
The Ω and B8-B9 loops of ADC-1 and ADC-1_TM_. (**A**) Superposition of monomer A from ADC-1 (pink) and ADC-1_TM_ (green). The β-hairpin-like conformation of the ordered Ω-loop in both structures is held together by two short antiparallel β-strands. (**B**) The same superposition for monomer B. Mutation of the phenylalanine to serine leads to a rearrangement of the main chain torsion angles (phi and psi) of the preceding residue (Arg321) by −26° and 56°, respectively. In both panels, residue labels for ADC-1_TM_ are italicized and colored orange. The three mutated residues are also colored light orange.

The crystal structures of ADC-1 and ADC-1_TM_ are isomorphous with the previously reported ADC-1 structure (PDB Code 4NET) and comprise a dimer in the asymmetric unit (Fig. S2A). Superposition of the ADC-1 and ADC-1_TM_ dimers onto 4NET showed no major structural differences between the three ADC-1 structures (Fig. S2A); however, localized differences between ADC-1 and ADC-1_TM_ are seen in the Ω-loop (strands B5a and B5b and helix A7a in the secondary structure numbering scheme given in reference 33) and the loop between strands B8 and B9 (Fig. 1).

The Ω-loop in monomer A of both enzymes adopts a β-hairpin-like conformation; however, the B8–B9 loop shows a large variation in conformation (Fig. 1A). In ADC-1, this loop bends outward from the active site, whereas in ADC-1_TM_, it folds markedly more inward. In monomer B of both structures, the B8–B9 loops adopt a similar conformation (different from that in monomer A) but are ~1.4 Å apart (Fig. 1B). This difference in location is a result of the Phe322Ser substitution in ADC-1_TM_, where the smaller serine side chain leads to rearrangement of the main chain of Arg321, whose side chain now projects in the opposite direction and forms hydrogen bonds with Asn204 and Gln207 of the Ω-loop. This results in the stabilization of this loop in ADC-1_TM_ (four residues disordered) relative to ADC-1 (13 residues disordered) (Fig. 1B; Fig. S2B). The proximity of the B8–B9 loop and the Ω-loop to the catalytic serine (Ser64) and the active site suggests that any conformational variations of these loops may affect substrate binding and/or catalysis.

**Fig 2:**
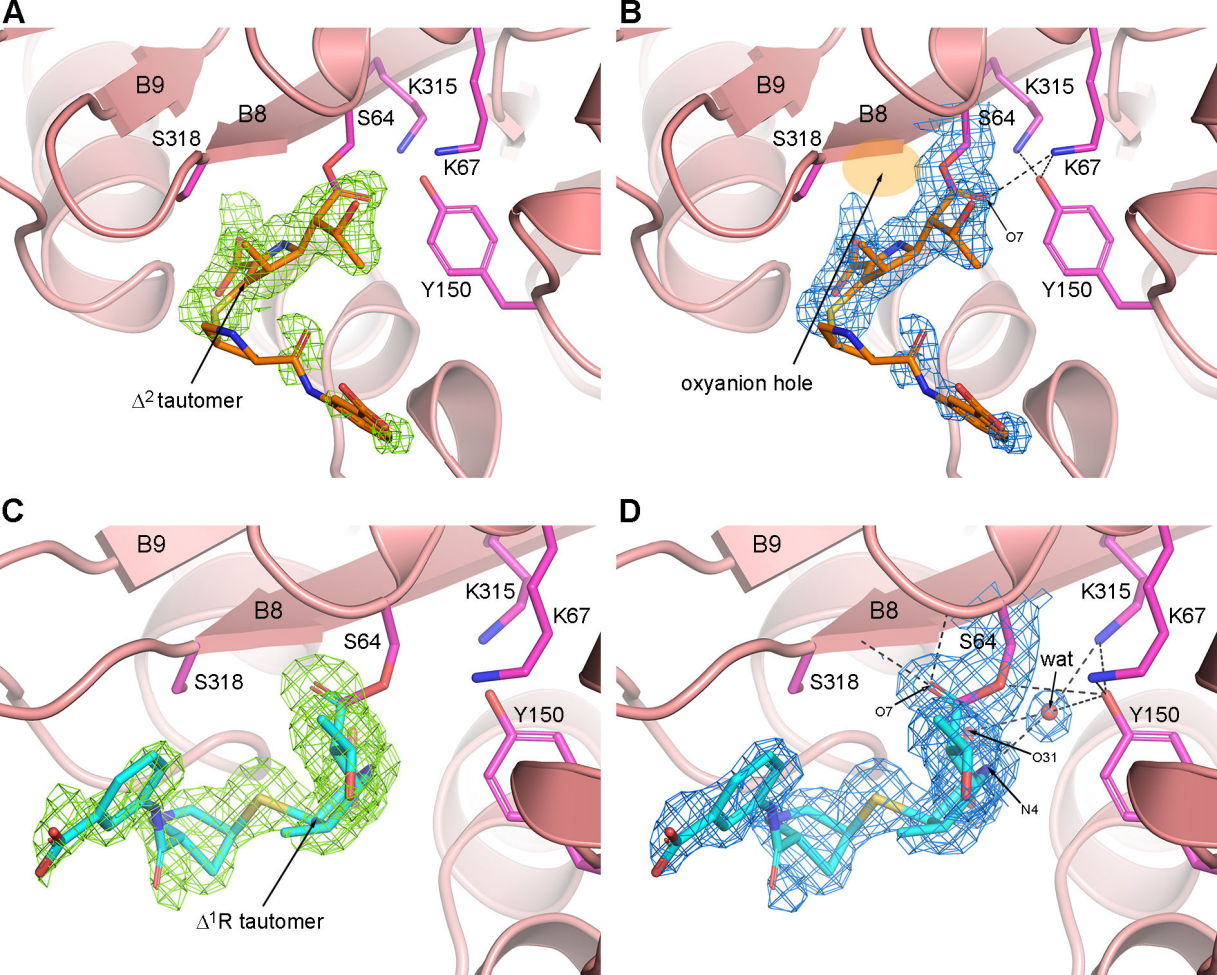
The ADC-1–ertapenem acyl–enzyme complex. (**A**) Polder omit density (green mesh, contoured at 3.5σ) for ertapenem (orange sticks) in monomer A. The pyrroline ring is well ordered, but the tail group beyond the sulfur shows a significant degree of disorder as evidenced by the patchy density. The pyrroline ring is in the Δ^2^ tautomeric conformation. (**B**) Final 2*F_o_ − F_c_* density (blue mesh, contoured at 1σ) for the bound carbapenem in monomer A, showing hydrogen bonds as black dashed lines. The location of the oxyanion hole is indicated by the orange ellipse. (**C**) Polder omit density (green mesh, contoured at 3.5σ) for ertapenem (cyan sticks) in monomer B shows a different binding mode for the acylated carbapenem. (**D**) Final 2*F_o_ − F_c_* density (blue mesh, contoured at 1σ) for the bound carbapenem in monomer B, where the O7 carbonyl is anchored in a canonical conformation in the oxyanion hole. A potential deacylating water is shown as a red sphere, 3.0 Å from the Oη of Tyr150, 2.6 Å from the Nζ of Lys315, and 3.2 Å from the main chain carbonyl oxygen of Thr316 (not shown for clarity).

The crystal structure of the ADC-1–ertapenem acyl–enzyme complex shows a clear dimorphism between the two monomers in the asymmetric unit (Fig. 2). In monomer A, the core of ertapenem is clearly defined, and the pyrroline ring is in the Δ^2^ tautomeric conformation with the exocyclic sulfur coplanar with the ring (Fig. 2A). The O7 carbonyl oxygen of ertapenem is directed away from the oxyanion hole (formed by the main chain amide nitrogen atoms of Ser64 and Ser318) where it is hydrogen bonded to Lys67 (Fig. 2B). This would prevent deacylation, as proposed earlier for the AmpC–imipenem complex (PDB code 1LL5) (34).

In contrast, in the ADC-1–ertapenem monomer B, the pyrroline ring is in the Δ^1^R tautomeric form (Fig. 2C), and the C7 carbonyl is bound in a canonical conformation in the oxyanion hole, hydrogen bonded to Ser64 and Ser318 (Fig. 2D). A potential deacylating water molecule is observed in the active site ~3.9 Å from the C7 atom of ertapenem, the point of nucleophilic attack leading to deacylation. However, this water is hydrogen bonded to two atoms of ertapenem (N4 and O31) (Fig. 2D), which could negatively impact its activation and also hinder a closer approach to the C7 atom. This, coupled with the Δ^1^R tautomeric configuration of ertapenem, which has been shown to be more resistant to hydrolysis (35), could hinder efficient deacylation from this complex. If both monomers A and B are also present in solution, only monomer B may contribute to the very slow deacylation of ertapenem detected kinetically.

### Molecular docking

**Fig 3:**
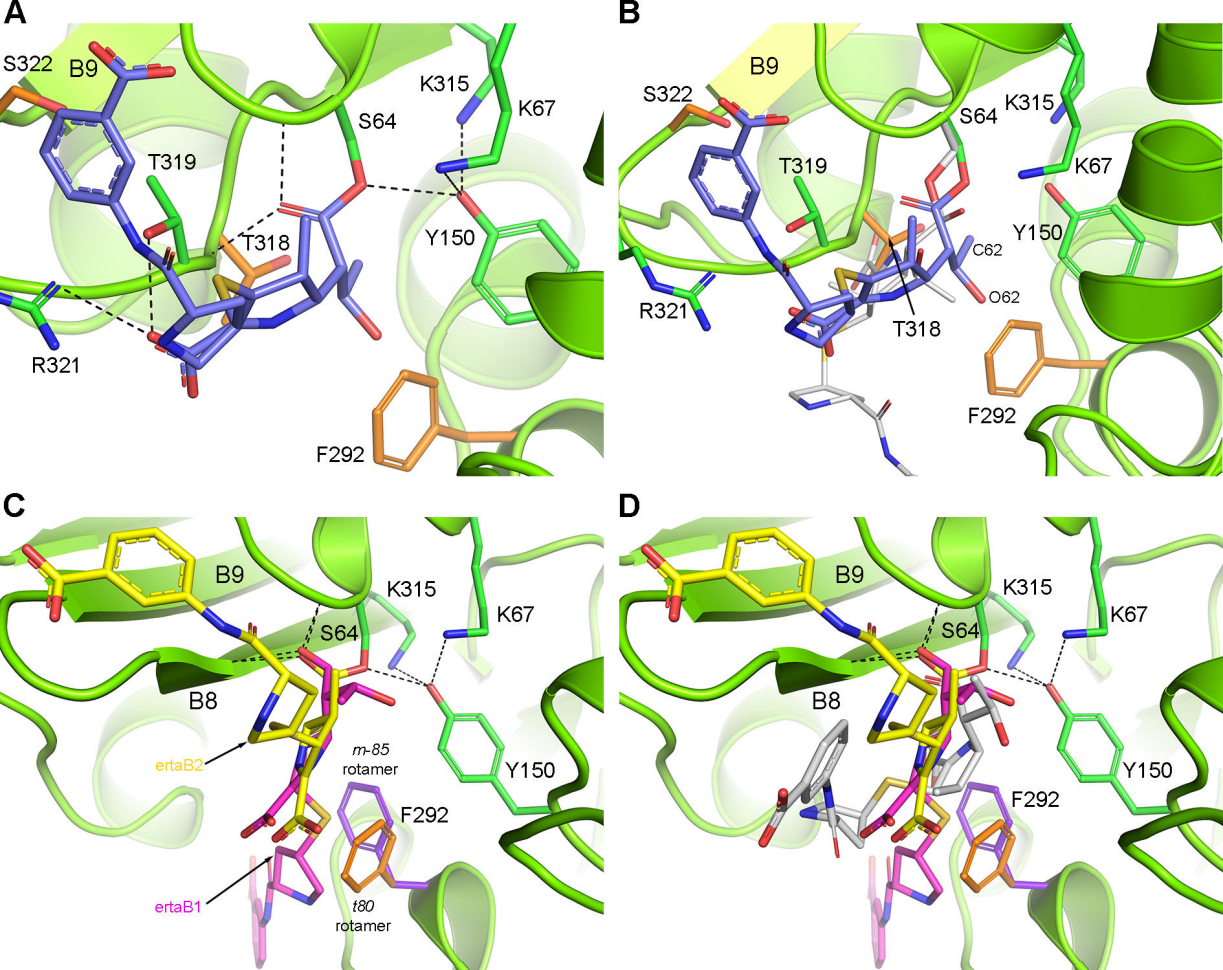
The docked ADC-1_TM_–ertapenem model. (**A**) Ertapenem (blue sticks) is docked into monomer A of ADC-1_TM_ (green ribbons and sticks, with mutated residues in orange). The C3 carboxylate makes hydrogen bonds with the side chains of Thr319 and Arg321, which are not present in the ADC-1 complex. The Arg321 side chain has moved ~2.8 Å further into the active site relative to ADC-1. (**B**) Superposition of ADC-1–ertapenem monomer A (carbapenem shown as gray sticks) onto the docked ADC-1_TM_–ertapenem complex monomer A (green ribbons and sticks, with mutated residues in orange). The side chain of Ser64 from the ADC-1–ertapenem crystal structure is shown as gray sticks. The rest of the ADC-1 structure is not shown for clarity. (**C**) Ertapenem docked into monomer B of ADC-1_TM_ in two conformations (ertaB1 and ertaB2), controlled by the dynamics of the Phe292 side chain. The Phe292 *m-85* rotamer (purple sticks) favors ertaB1 (magenta sticks), and the Phe292 *t80* rotamer (orange sticks) favors ertaB2 (yellow sticks). (**D**) Monomer B of ADC-1_TM_ with the two docked ertapenem conformers (magenta and yellow sticks) and ertapenem from monomer B of the ADC-1 crystal structure (gray sticks) superimposed.

Since soaking experiments with ADC-1_TM_ and ertapenem did not capture an acyl–enzyme complex, molecular docking was carried out to generate models of the complexes. The three different tautomerization states of the acylated carbapenem (Δ^1^R, Δ^1^S, and Δ^2^) were tested in each monomer. The side chains of Phe292 and Thr318 were allowed to be flexible during the simulations since these two mutated residues are directed inward and change the structure of the active site relative to ADC-1. In monomer A, the Δ^2^ tautomer consistently docked with the highest docking scores, and a consistent set of poses was obtained where the O7 carbonyl oxygen was directed into the oxyanion hole (Fig. 3A), in contrast to monomer A of the ADC-1–ertapenem crystal structure (Fig. 2B and 3B). In the monomer A docked poses, the core of ertapenem is substantially shifted relative to its position in the crystal structure (Fig. 3B), with the pyrroline ring rotated by ~60° and translated ~4 Å away from strand B8. This movement of ertapenem is a result of the difference in conformation of the B8–B9 loop in ADC-1_TM_, which places the side chain of Thr318 (one of the three mutations) into a position formerly occupied by the C3 carboxylate in ADC-1 (Fig. 3B). As a result, none of the hydrogen bonding interactions observed in monomer A of the ADC-1–ertapenem complex are retained in the docked pose.

Unlike in monomer A, ertapenem in monomer B docked with the highest scores only in the Δ^1^R tautomerization state (as observed in the ADC-1 structure), with the O7 carbonyl in the oxyanion hole. Ertapenem was observed in two orientations (ertaB1 and ertaB2) of roughly equal energy (Fig. 3C), both of which are non-canonical compared to monomer B of the ADC-1–ertapenem crystal structure (Fig. 3D). The switch between these two orientations is facilitated by a rotation of the Phe292 side chain from the *t80* rotamer to the more preferred *m-85* rotamer, allowing the entire ertapenem molecule to rotate almost 180°.

It is tempting to suggest that the repositioning of the C7 carbonyl into the oxyanion hole in monomer A resembles the more canonical conformation of the acyl bond, and it is this structural change that results in the enhanced deacylation rate of ADC-1_TM_. However, to test the stability and viability of docked complexes and to gain a fuller understanding of how the reorientation of the acyl-enzyme intermediate in both monomers could give rise to altered kinetics in the mutant, we undertook molecular dynamics (MD) simulations on the complexes.

### MD simulations

Triplicate 100-ns MD simulations were conducted for the docked ADC-1_TM_–ertapenem models in both monomers (designated ADC-1_TM_–ertaA, ADC-1_TM_–ertaB1, and ADC-1_TM_–ertaB2). Similar simulations were also performed on the individual monomers of the ADC-1-ertapenem crystal structure (designated ADC-1–ertaA and ADC-1–ertaB) for comparison. The root mean square deviations for the protein main chains relative to the initial models were between 1.5 and 2.0 Å for all complexes, indicating that the simulations were stable. For deacylation, a water molecule must come into the active site in the vicinity of the Tyr150 side chain and the scissile acyl bond in a deacylating water pocket (DWP) delineated by the side chains of Ser64, Tyr150, and Lys315, and the Thr316 main chain carbonyl oxygen (Fig. S3A). The presence of water in this DWP was monitored over the duration of the MD simulations. Radial distribution functions (RDFs) of all the solvent molecules relative to a point representing the geometric center (CofG) between these four residues (Fig. S3B) in the MD trajectories of the five structural models were calculated. A water molecule would need to come to within at least 2 Å of this point to be oriented at the correct distance and angle for efficient nucleophilic attack on the C7 atom.

**Fig 4:**
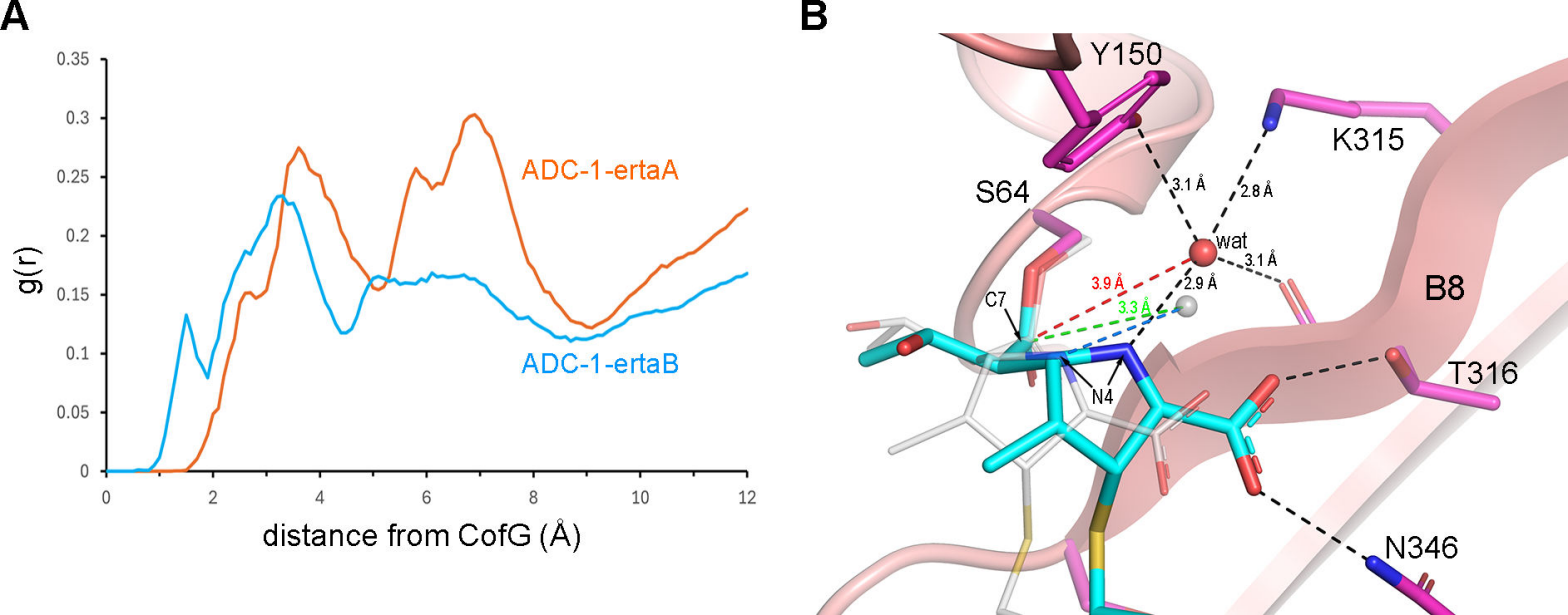
Analysis of MD simulations of the ADC-1–erta complexes. (**A**) RDF showing the probability distribution [g(r)] of water molecules as a function of distance to the CofG for monomers A (orange trace) and B (cyan trace) in the 100-ns MD simulation of the ADC-1–ertapenem crystal structure. (**B**) Representative frame from the ADC-1–ertaB MD simulation showing a water molecule (wat) in the DWP, hydrogen bonded (black dashed lines) to Tyr150, Lys315, Thr316, and the N4 atom of ertapenem (cyan sticks). The distance between wat and the C7 atom of ertapenem is ~3.9 Å (red dashed line). A frame from the simulation where the water (small gray semi-transparent sphere) was at its closest approach (3.3 Å, green dashed line) to the C7 atom was superimposed. This close approach results from the loss of hydrogen bonds between the protein (Thr316 and Asn346) and the carboxylate of ertapenem (thin gray sticks), and movement of the carbapenem by 2–3 Å away from strand B8. The hydrogen bond to the N4 atom of the ertapenem is maintained (blue dashed line).

The RDF for ADC-1–ertaA (Fig. 4A) and inspection of the MD trajectory revealed that no water molecules came into the DWP close enough to facilitate deacylation. This likely results from the restriction of water access to the DWP by the O7 oxygen of ertapenem (Fig. 2B). These data indicate that ertapenem bound in this orientation, albeit in the more favored Δ^2^ tautomerization state, would most likely not be susceptible to deacylation from the complex. In the RDF for ADC-1–ertaB (Fig. 4A), a peak at ~1.8 Å suggests the presence of some water molecules in the vicinity of the scissile bond. Inspection of the ADC-1–ertaB MD trajectory showed that more than a dozen water molecules came into the DWP over the duration of the simulation. The hydrogen bonding interactions between the water molecules and the residues lining the DWP, along with the N4 and C7 atoms of the acyl–enzyme complex, were monitored, and the plots of these distances for a representative water molecule are shown in Fig. S4A through C. A frame from the MD trajectory for this water molecule is given in Fig. 4B and shows that while this water molecule maintains interactions with the residues lining the DWP, it is ~4.4 Å on average from the C7 atom (Fig. S4D). This is significantly longer than the van der Waals non-bonded contact distance (3.22 Å for a carbon–oxygen pair) (36) required for efficient nucleophilic attack on the scissile bond. The water is prevented from getting closer to the C7 atom by the N4 atom of the pyrroline ring, which is anchored in place by hydrogen bonds between the C3 carboxylate and the Thr316 and Asn346 side chains (Fig. 4B). Several frames showed that water molecules approach the C7 atom to within 3.3 Å (Fig. S4D; Fig. 4B). This is facilitated by a loss of contact between the C3 carboxylate and the Thr316 and Asn346 side chains, and the subsequent outward swing of ertapenem from the active site (Fig. 4B). This rearrangement of the complex is very short lived and reverts to its original conformation after 10–20 ps, with the water molecule pushed back further into the DWP by steric pressure from the N4 atom. These data suggest that while deacylation could occur from the ADC-1–ertaB complex during dynamic changes like this, it would proceed rather inefficiently, consistent with the very slow deacylation observed kinetically.

**Fig 5:**
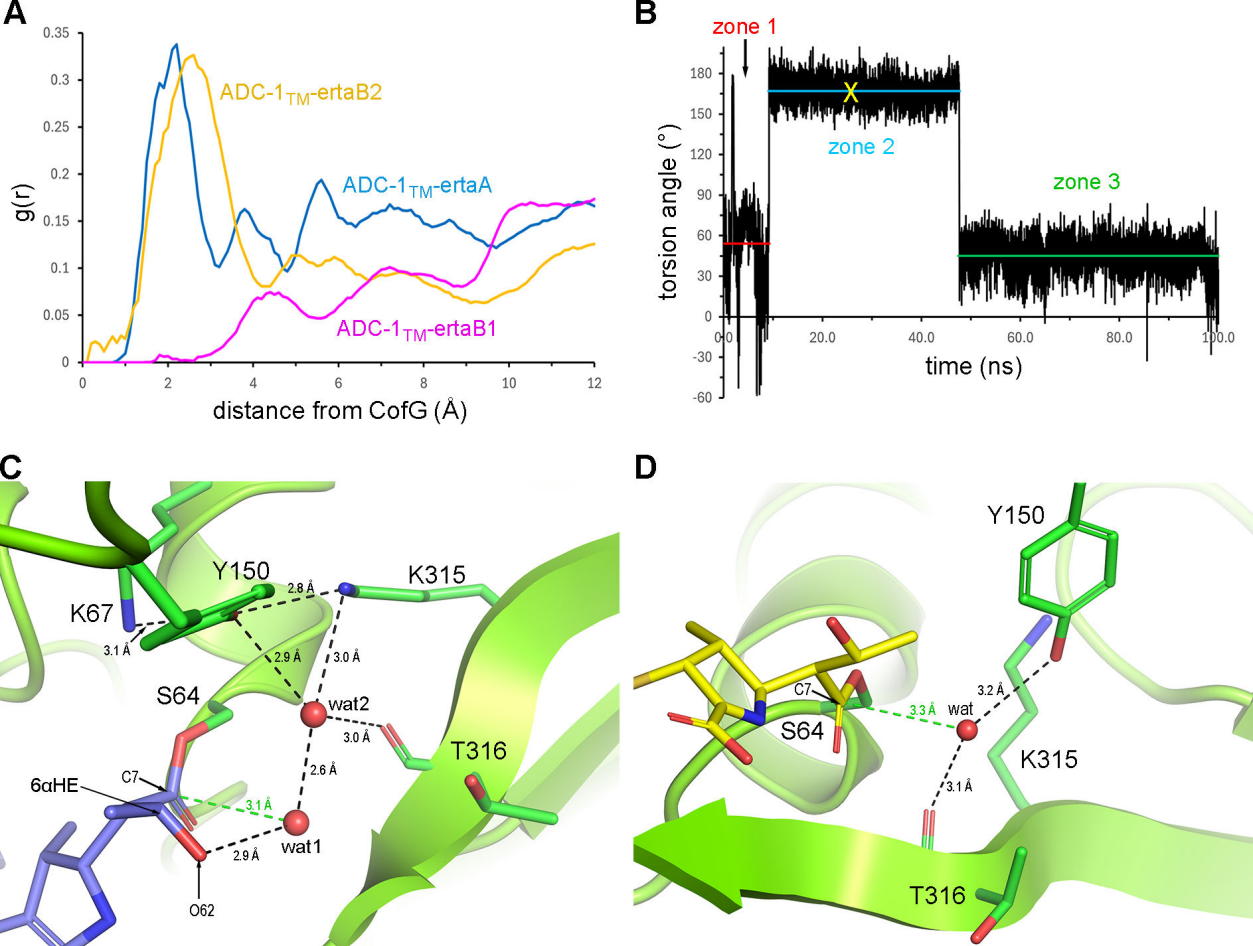
Analysis of MD simulations of the ADC-1_TM_–erta docked complexes. (**A**) RDF showing the probability distribution of water molecules as a function of distance to the CofG for monomers A (blue trace) and B (magenta and yellow traces) in the 100-ns MD simulation of the ADC-1_TM_–ertapenem docked models. (**B**) Plot of the 6αHE torsion angle for the ADC-1_TM_–ertaA MD simulation. Three zones are indicated, with the red, cyan, and green lines indicating the average torsion angle for each zone. (**C**) Representative frame from the ADC-1_TM_–ertaA MD simulation (the yellow X in panel B indicates the location of this frame). A water molecule (wat1) is hydrogen bonded to the O62 atom of the 6αHE group of ertapenem (blue sticks) and is well aligned for nucleophilic attack of the scissile bond (green dashed line). The Tyr150 side chain is anchored by interactions with Lys67 and Lys315. A second water (wat2) bridges between Tyr150 and wat1, providing a proton shuttle for the activation of wat1. (**D**) Representative frame from the ADC-1_TM_–ertaB2 MD simulation. A water molecule (wat) is hydrogen bonded to Tyr150 and Thr316 and comes within 3.3 Å (green dashed line) of the C7 atom of ertapenem (yellow sticks).

In the case of the mutant enzyme, the RDFs for ADC-1_TM_–ertaA and ADC-1_TM_–ertaB2 both have strong peaks at ~2 Å (Fig. 5A), highly suggestive of prolonged occupation of the DWP in both monomers. In contrast, the RDF for ADC-1_TM_–ertaB1 (Fig. 5A) shows a smaller peak at ~4 Å, indicative of water molecules outside the DWP where they would be unlikely to perform deacylation. In ADC-1_TM_–ertaA, although a number of water molecules enter the DWP, their ability to come close enough to the C7 atom is governed by the rotameric state of the 6α-hydroxyethyl (6αHE) group of ertapenem (Fig. 5B). This group is initially in what is deemed an inward-facing rotameric conformation (zone 1), whereby both the C62 and O62 atoms are in the DWP pointed toward Tyr150 (Fig. 3A). Monitoring of the 6αHE torsion angle over the duration of the MD simulation (Fig. 5B) showed that this group rotates ~120° to a more outward-facing conformation (zone 2) after 9.2 ns, where the O62 atom remains on the edge of the DWP, and the C62 atom points out into the active site. This opens the pocket, and most of the water molecules come into the DWP while the 6αHE is in this orientation. A representative water molecule (wat1) from zone 2 (Fig. 5C) is hydrogen bonded to the O62 atom and comes within ~3.1 Å of the C7 atom at an angle almost perpendicular to the plane of the acyl bond, conducive to efficient nucleophilic attack. This water is over 5 Å from the Tyr150 side chain, too far away to be activated. However, every time wat1 enters the site, another water molecule (wat2; Fig. 5C) invariably enters and bridges between wat1, Tyr150, and Lys315, and we propose that wat2 acts as a proton shuttle facilitating the activation of wat1 in a novel deacylation mechanism specific to this mutant complex. After another ~40 ns, the 6αHE rotates back to the original inward-facing conformation (zone 3; Fig. 5B), and from this point onward, very few water molecules are able to enter.

The contrasting RDFs for the two ADC-1_TM_ monomer B docked complexes (Fig. 5A) suggest that the DWP is accessible only in the ADC-1_TM_–ertaB2 complex. Indeed, an inspection of the ADC-1_TM_–ertaB1 MD trajectory showed no water molecules coming within 5 Å of the scissile bond (Fig. S5A), and this is due to complete blockage of the DWP by the methyl group (C11) on the pyrroline ring (Fig. S5B) for the entire 100-ns simulation (Fig. S5C). Since deacylation from this complex is highly unlikely, further discussion will be limited to the ADC-1_TM_–ertaB2 complex. In this complex, water molecules come into the DWP and form hydrogen bonds with Tyr150 (Fig. S5D). The Lys315 side chain maintains an interaction with Tyr150, but a hydrogen bond between the lysine and water is rarely seen (Fig. S5D). During the MD simulation, Tyr150 loses contact with Lys67 and swings deeper into the pocket by ~2.5 Å (Fig. 5D). The loss of these hydrogen bonds, the structural changes in the active site observed in the docking simulations, and conformational changes of ertapenem result in a more open and dynamic DWP. This allows water molecules to approach closer to the scissile bond (~4.1 Å on average) compared to the ADC-1–ertaB complex, with several coming much closer (Fig. S5E and F) and aligning with a more optimal angle of attack (data not shown). This is due to the alternative binding of ertapenem such that the pyrroline ring is out of the DWP (Fig. 5D). This arrangement would allow more efficient deacylation from this complex compared to ADC-1–ertaB (Fig. 4B).

Comparison of the three complexes from which deacylation is probable showed that the mutational changes of ADC-1_TM_ result in changes in both monomers of the orientation of the bound ertapenem and the environment around Tyr150, which play a key role in deacylation (Fig. S6A). In the ADC-1–ertaB complex, for most of the MD trajectory, the DWP remains constricted by the incursion of the pyrroline ring of ertapenem. The ring swings away from the pocket only intermittently (Fig. 4B), suggesting that only some deacylation could proceed. In both of the ADC-1_TM_–erta complexes, ertapenem is in two alternative positions, both of which would allow for better deacylation than that observed for ADC-1–erta. In the ADC-1_TM_–ertaA complex, the 6αHE group of ertapenem partially occludes the DWP; however, its rotation allows a deacylating water to occupy a position ideally aligned for nucleophilic attack on the C7 atom (Fig. 5C; Fig. S6B). In the ADC-1_TM_–ertaB2 complex, ertapenem does not occlude the DWP, and Tyr150 is 2.5 Å closer to the scissile bond relative to ADC-1–ertaB. This allows water molecules to become activated by Tyr150 for deacylation as they approach the C7 atom. These MD simulations, combined with the MIC, kinetic, and structural data, provide insights into the molecular mechanisms by which the three amino acid substitutions in ADC-1 significantly increase the deacylation rate of ertapenem, thus contributing to the carbapenemase activity of the mutant enzyme.

## MATERIALS AND METHODS

### Strains and plasmids

To maintain plasmids, bacteria were grown at 37°C in Luria-Bertani (LB) broth in the presence of either 30 µg/mL (for *A. baumannii* ATCC 17978) or 60 µg/mL [for *E. coli* DH5α and BL21(DE3)] kanamycin. Cloning of the *bla*_ADC-1_ gene into the *A. baumannii–E. coli* shuttle vector pNT255, pHF016, and pET24a(+) was previously described (28, 33). The pNT255 shuttle vector was constructed in our lab and contains the ISAba1 promoter of *A. baumannii* for gene expression and also two origins of replication, one from the *E. coli* plasmid pBR322 and another from the *Acinetobacter calcoaceticus* vector pWH1266, which allow pNT255 to be transferred interchangeably between *E. coli* and *A. baumannii*. Single mutations and their combinations in the *bla*_ADC-1_ gene were generated by site-directed mutagenesis using the primers listed in Table S1. The gene encoding ADC-68 under its original signal peptide (GenBank accession no. KC866352.1) was custom synthesized (GenScript) and cloned into pNT255. For large-scale expression of ADC-68, the first 69 bases encoding the signal peptide were removed by PCR amplification, and the gene was cloned into pET24a(+) and expressed in *E. coli* BL21 (DE3). Nucleotide sequences of all constructs were verified (McLab).

### Selection of ADC-1 mutants

ADC-1 mutants were obtained by four successive serial passage steps of *E. coli* DH5α harboring the pHF016 plasmid with the *E. coli*-optimized *bla*_ADC-1_ gene under the OmpA leader (1 × 10^7^ CFU/mL) in 300 mL of LB media containing imipenem or ertapenem (two- to fourfold above the MIC of 0.25 or 0.015 µg/mL, respectively) until the cultures became turbid, which generally took 1–2 days. After the first passage, bacterial growth was observed only in media containing ertapenem; thus, subsequent rounds of selection were performed with this antibiotic. After another three cycles, growth was observed up to 0.5 µg/mL of ertapenem. Subsequently, bacteria were plated onto LB agar containing 0.25 µg/mL of ertapenem. Plasmid DNA from two dozen individual colonies was isolated, and the *bla*_ADC-1_ gene was sequenced, which identified up to eight amino acid substitutions. To narrow down which of these mutations was essential for resistance to ertapenem, equal aliquots of DNA from all colonies were mixed and back shuffled with the DNA encoding the parental ADC-1 enzyme in a 5:95 ratio, respectively, as previously described (37). Following shuffling, the gene library was cloned into pHF016, introduced into *E. coli* DH5α, and plated onto LB agar containing 0.25 µg/mL of ertapenem. Plasmid DNA from two dozen individual colonies was sequenced. The clone producing the highest level of resistance and encoding the lowest number of amino acid substitutions (Val292Phe, Ser318Thr, and Phe322Ser) was further studied.

### Antibiotic susceptibility testing

MICs of β-lactams against *E. coli* DH5α and *A. baumannii* ATCC 17978 were determined in triplicate using the broth microdilution method according to the Clinical and Laboratory Standards Institute (CLSI) guidelines (38).

### Growth curves

Overnight cultures of *A. baumannii* ATCC 17978 expressing either ADC-1 or ADC-1_TM_ were diluted 1:100 in Mueller–Hinton medium supplemented with 30 µg/mL of kanamycin. At an optical density (OD_600_) of 0.2, the cultures were further diluted 200-fold into the same medium and grown at 37°C with constant agitation. The OD_600_ of aliquots from the cultures was monitored every hour and plotted versus time on a semi-log scale. Three independent experiments were carried out to generate the growth curves.

### Protein expression and purification

ADC-1 and ADC-68 were expressed and purified as previously described (33). For the ADC-1 triple mutant, an additional purification step was required. The enzyme was dialyzed against 20 mM Tris, pH 7, and loaded onto a DEAE anion-exchange column (Bio-Rad). The protein was eluted in the flow-through fraction.

### Enzyme kinetics

Data were collected in at least triplicate at 22°C using a Cary60 spectrophotometer (Agilent) or an SFM-300 stopped-flow instrument (Bio-Logic). Reactions containing various concentrations of β-lactams (27, 33) in 50 mM sodium phosphate, pH 7.4, were initiated with the addition of enzyme and monitored for up to 60 min. For ADC-1, reactions with carbapenems, which were poor substrates, contained 2–200 µM antibiotics and 0.2–4 µM enzyme, while reactions with the good substrate ampicillin contained 10–75 µM β-lactam and 27–82 nM enzyme. For ADC-1_TM_, reactions with the poor substrate imipenem contained 2–100 µM antibiotic and 0.2–5 µM enzyme, while reactions with the other three carbapenems and ampicillin, which were better substrates, contained 2–100 µM β-lactams and 200–500 nM enzyme. For ADC-68, reactions contained 2–200 µM carbapenems, which were poor substrates, and 0.2–1 µM enzyme, while reactions with the good substrate ampicillin contained 10–40 µM antibiotic and 12.5–25 nM enzyme. Control reactions in the absence of enzymes were performed to monitor the nonspecific hydrolysis of the carbapenems. In all cases, there was a significant difference (≥ninefold) between the slope of the reaction and that of the control. The steady-state velocities were obtained from the linear portion of the progress curves and plotted versus the concentration of β-lactam. The data were fitted to the Michaelis–Menten equation to determine the parameters *k*_cat_ and *K*_*m*_ using nonlinear regression in Prism 10 (GraphPad Software Inc.).

To determine the acylation rate constant, *k*_2_, reactions containing 10 µM ertapenem in 50 mM sodium phosphate, pH 7.4, were initiated by the addition of excess ADC-1 or ADC-1_TM_ (20–100 µM). Progress curves were analyzed as previously described (27). Jump dilution experiments were attempted to empirically determine the deacylation rate constant, *k*_3_, for ADC-1 and ADC-1_TM_, where each enzyme (4–20 nM) was preincubated with ertapenem (2–10 µM) in 50 mM sodium phosphate, pH 7.4, containing 0.04 mg/mL of bovine serum albumin (BSA) for 1 min prior to diluting either 20- or 100-fold into nitrocefin (600 µM for ADC-1 and 150 µM for ADC-1_TM_; λ = 500 nm and Δ*ε* = +15,900 M^−1^ cm^−1^) containing 0.04 mg/mL of BSA. Control reactions without ertapenem were performed under the same conditions. Progress curves were analyzed as previously described (27). The *k*_3_ values were also calculated using the *k*_2_ and *k*_cat_ values according to the relationship *k*_3_ = (*k*_cat_ × *k*_2_)/(*k*_2_ − *k*_cat_).

### Crystallization and data collection

ADC-1 and ADC-1_TM_ were crystallized by sitting-drop at 15°C in Intelli-plates (Hampton Research). Drops comprising 1 µL of reservoir solution (0.2 M lithium chloride, 20% PEG 3350) were mixed with 1 µL of protein solution at a concentration of 25 mg/mL. Apo enzyme crystals were swished through the crystallization solution augmented with 30% glycerol prior to flash cooling in liquid nitrogen. Diffraction data for ADC-1 and ADC-1_TM_ were collected at SSRL beamline BL9-2 using X-rays at 13,000 eV (0.9537 Å) with a Pilatus 6M detector. Data were processed and scaled with XDS (39) and AIMLESS (40) to give a final data set with resolutions of 1.3 Å for ADC-1 and 1.8 Å for ADC-1_TM_ (Table S2).

Soaking experiments with ertapenem were performed on ADC-1 and ADC-1_TM_ crystals. The ADC-1–ertapenem crystals diffracted ~1.6 Å, and a data set was collected at BL9-2 using X-rays at 12,658 eV (0.9795 Å). The data were processed as for ADC-1 (Table S2). The ADC-1_TM_ crystals proved to be very sensitive during soaking, dissolving almost immediately, and no usable diffraction data could be collected.

The ADC-1 and ADC-1_TM_ structures were solved by molecular replacement using the previously reported ADC-1 structure (PDB code 4NET) (33) as the search model. The ADC-1–ertapenem structure was solved by molecular substitution using the partially refined ADC-1 structure. All structures comprised two monomers in the asymmetric unit.

### Molecular docking and MD simulations

Ertapenem was docked into both monomers of ADC-1_TM_ using ICM-Pro v3.9-3a (41, 42). The three possible tautomeric states (Δ^1^R, Δ^1^S, and Δ^2^) were tested in both monomers. Simulations were performed six times, and the most consistent poses with the highest (most negative) ICM score were output as PDB files for further analysis.

MD simulations were performed in triplicate for 100 ns on the ADC-1–ertaA and ADC-1–ertaB crystal structures, and the ADC-1_TM_–ertaA, ADC-1_TM_–ertaB1, and ADC-1_TM_–ertaB2 models derived from docking. The simulations were performed using Desmond (43) in the Schrodinger 2019–2 release as described previously (28, 29). Briefly, Maestro (Schrodinger) was used to add the covalent linkage between the C7 atom and the Oγ of Ser64, parameterize the ligands, protonate the complex using EPIK, and assign hydrogen bonds. The model systems were prepared using the OPLS3e force field and minimized prior to the addition of the pre-defined TIP3P water model. The overall charges of the complexes were neutralized with Na^+^ and Cl ^−^ ions, and 0.15 M salt (NaCl) was added throughout the simulation box, although excluded from a sphere of radius 20 Å from the ligands. The final 100-ns production steps were run at 1 atm pressure and 300 K using the Martyna–Tuckerman–Klein chain coupling scheme with a coupling constant of 2.0 ps for pressure control and the Nosé–Hoover chain coupling scheme for temperature control. Nonbonded forces were calculated using an r-RESPA integrator. The trajectories were saved at 10-ps intervals, and subsequent analyses were carried out in Maestro. Maestro and Desmond were run on the SHERLOCK 3.0 HPC cluster at Stanford University.

## Data Availability

The ADC-1, ADC-1_TM_, and ADC-1–ertapenem structure factors and atomic coordinates have been deposited in the PDB with PDB codes 9MTU, 9MTV, and 9MTW, respectively.

## SUPPLEMENTAL MATERIAL

Supplemental material mbio.00185-25-s0001.pdf Fig. S1 to S6; Tables S1 and S2.

ASM does not own the copyrights to Supplemental Material that may be linked to, or accessed through, an article. The authors have granted ASM a non-exclusive, world-wide license to publish the Supplemental Material files. Please contact the corresponding author directly for reuse.
