# Supplementary material for: Evolution of carbapenemase activity in the class C β-lactamase ADC-1
Source: mBio. 2025 Apr 28;16(6):e00185-25. doi: 10.1128/mbio.00185-25 (PMC12153350; doi:10.1128/mbio.00185-25)
Supplement: Supplemental material — Fig. S1 to S6; Tables S1 and S2. [file mbio.00185-25-s0001.pdf]

Supporting Information for

Evolution of Carbapenemase Activity in the Class C β-Lactamase ADC-1

Nichole K. Stewart[a], Marta Toth[a], Monolekha Bhattacharya[a,*], Clyde A. Smith[b,c,#], and Sergei B. Vakulenko[a,#]

[a]Department of Chemistry and Biochemistry, University of Notre Dame, Notre Dame, IN 46556, USA

[b]Stanford Synchrotron Radiation Lightsource, Stanford University, Menlo Park, CA 94025, USA

[c]Department of Chemistry, Stanford University, Stanford, CA 94305, USA

Running Title: ADC carbapenemase

[#]Address correspondence to:

Prof. Sergei B. Vakulenko, ph: 574-631-2935, Fax: 574-631-6652, E-mail: svakulen@nd.edu

Dr. Clyde A. Smith, ph: 650-926-8544, Fax: 650-926-3292, E-mail: csmith@slac.stanford.edu

*Present address: Monolekha Bhattacharya, Serotonin Labs India Pvt. Ltd, Bangalore, Karnataka 560076, India

Table S1. Primers used to generate ADC-1 mutants

Primer Name and Sequence (5' to 3')[a]	Mutant(s) Produced
oNS18-pho[b] TTT ATG AAA CCT AAT AAA GTG ACT GCT ATT TC<br>oNS03 AAT CTG TTC TGA ATT ACT GTC TAA TAA AGT TTG TAA CGT TGC C	Val292Phe
oNS04 G TAC CAT AAA ACT GGC ACA ACT AAC CGT TTC GGA AC<br>oNS05 GT TCC GAA ACG GTT AGT TGT GCC AGT TTT ATG GTA C	Ser318Thr
oNS19-pho TCT GGA ACC TAT GTG GTG TTT ATT CCT AAA G<br>oNS20 ACG GTT AGT TGA GCC AGT TTT ATG GTA C	Phe322Ser
oNS19-pho TCT GGA ACC TAT GTG GTG TTT ATT CCT AAA G<br>oNS21 ACG GTT AGT TGT GCC AGT TTT ATG GTA C	Ser318Thr/Phe322Ser

[a]Red letters indicate the mutation relative to the parental $bla_{ADC\text{-}1}$ gene sequence.

[b]Pho, phosphorylated at the 5' terminus.

Table S2. Data collection and refinement statistics[a]

		ADC-1	ADC-1$_{TM}$	ADC-1-ertapenem
Data Collection				
Space group		$P2_1$	$P2_1$	$P2_1$
Unit cell, a, b, c (Å), β (°)		43.1, 182.0, 50.3, 99.4	43.1, 182.2, 50.8, 99.4	43.0, 183.1, 50.5, 99.5
Resolution (Å)		38.6-1.30 (1.32-1.30)	38.6-1.80 (1.84-1.80)	38.6-1.60 (1.63-1.60)
Reflections	- observed	1261095	489125	403515
	- unique	179263	70428	99666
R_{meas}[b]		0.078 (1.15)	0.120 (1.35)	0.80 (1.16)
R_{pim}[c]		0.029 (0.447)	0.045 (0.516)	0.039 (0.577)
$I / \sigma I$		12.4 (1.4)	9.2 (1.3)	9.8 (1.3)
Completeness (%)		96.0 (89.2)	98.7 (93.7)	98.6 (96.9)
CC½[d]		0.999 (0.718)	0.997 (0.676)	0.997 (0.491)
Average multiplicity		7.0 (6.3)	6.9 (6.9)	4.0 (4.0)
Wilson B (Å^2)		14.7	27.6	23.7
Refinement				
PDB Code		9MTU	9MTV	9MTW
R_{work} / R_{free}[e]		0.1520 / 0.1880	0.1872 / 0.2149	0.1825 / 0.2269
Reflections, work/free		179059 / 9081	70294 / 3399	99491 / 4946
Number of atoms	- protein	5679	5698	5623
	- water	602	307	396
	- ligands	-	-	66
B-factors (Å^2)	- protein	21.2	32.2	28.4
	- water	32.3	33.3	29.7
	- ligands	-	-	33.7
rms deviations	- bond lengths (Å)	0.005	0.07	0.06
	- bond angles (º)	0.79	0.84	0.98
Ramachandran plot[f]	- favored (%)	97.4	97.2	98.0
	- outliers	0	2	0
Molprobity Score[f]		1.21 (95th percentile)	1.34 (98th percentile)	1.03 (100th percentile)
Molprobity Clashscore[f]		2.1	3.59	2.46

[a] Numbers in parentheses refer to the highest resolution shell.
[b] R_{meas} is the redundancy-independent merging R factor (1).
[c] R_{pim} is the precision-indicating merging R factor (1).
[d] Correlation between intensities from random half-sets of data (2).
[e] R_{free} was calculated using a test set comprising 5% of the data.
[f] Calculated with the program MOLPROBITY (3).

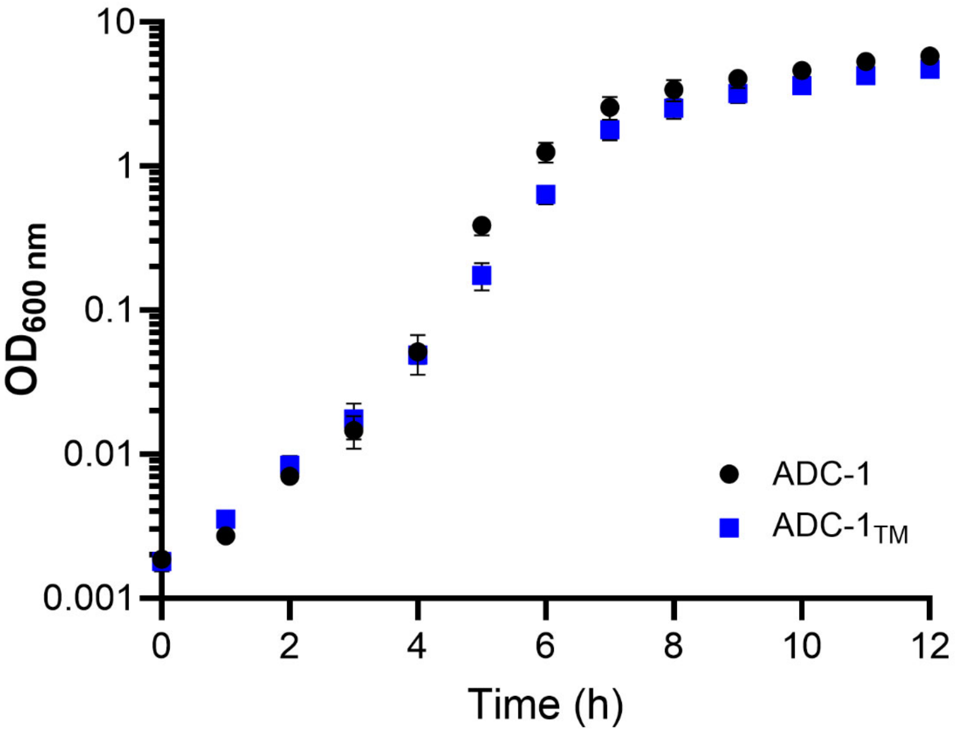

Figure S1. Growth curves of *A. baumannii* ATCC 17978 producing ADC-1 or ADC-1$_{TM}$.

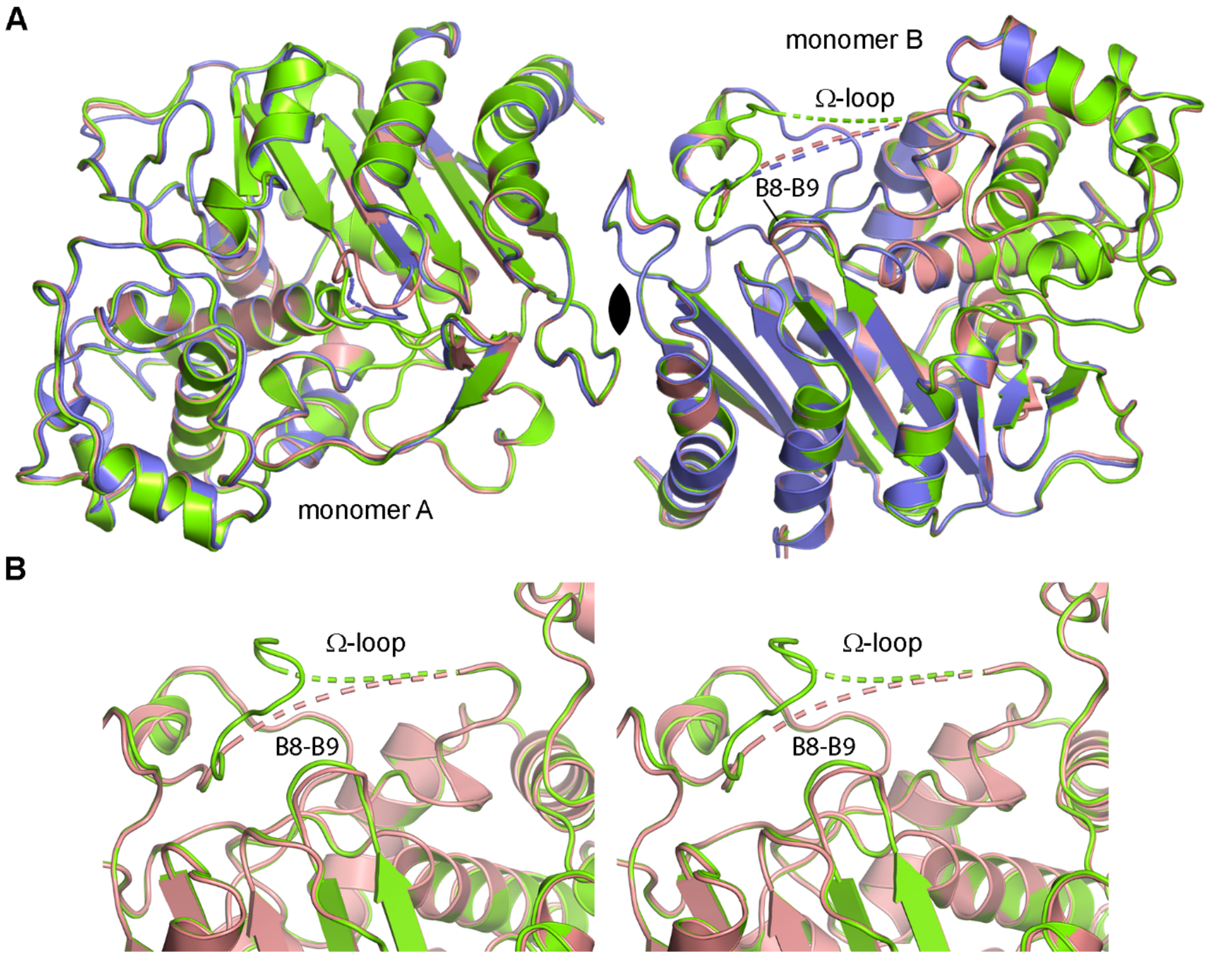

Figure S2. The ADC-1 crystal structures. (A) Superposition of ADC-1 (pink) and ADC-1_{TM} (green) onto 4NET (blue) gave *rmsd*s of 0.36 Å and 0.46 Å, respectively. The two independent molecules in the asymmetric unit are labeled monomer A and monomer B. The approximate location of the non-crystallographic two-fold axis is indicated by the black lens-shaped symbol. **(B)** Closeup of monomer B showing the disorder in the Ω-loop of ADC-1 (pink) and ADC-1_{TM} (green).

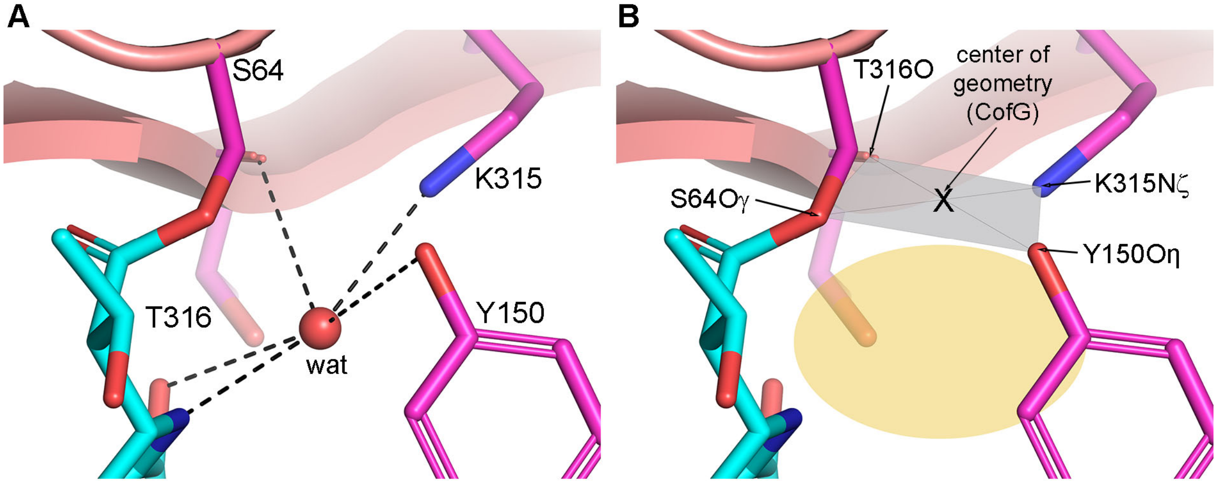

Figure S3. The deacylating water pocket (DWP) in ADC-1-ertaB. (A) The deacylating water (wat) in monomer B of the ADC-1-ertapenem crystal structure, showing the hydrogen bonding interactions (black dashed lines). **(B)** Location of the center of geometry (CofG; X) on a plane between the Ser64 Oγ atom, the Tyr150 Oη atom, the Lys315 Nζ atom, and the main chain oxygen atom of Thr316. Equivalent positions in the other three models used for MD simulations were constructed. The CofG was used to generate the RDFs shown in Figures 4A and 5A. Water molecules deemed to be in the DWP and capable of nucleophilic attack on the acyl bond would need to come within 2-3 Å of the CofG and be located in a region represented by the semi-transparent orange ellipsoid.

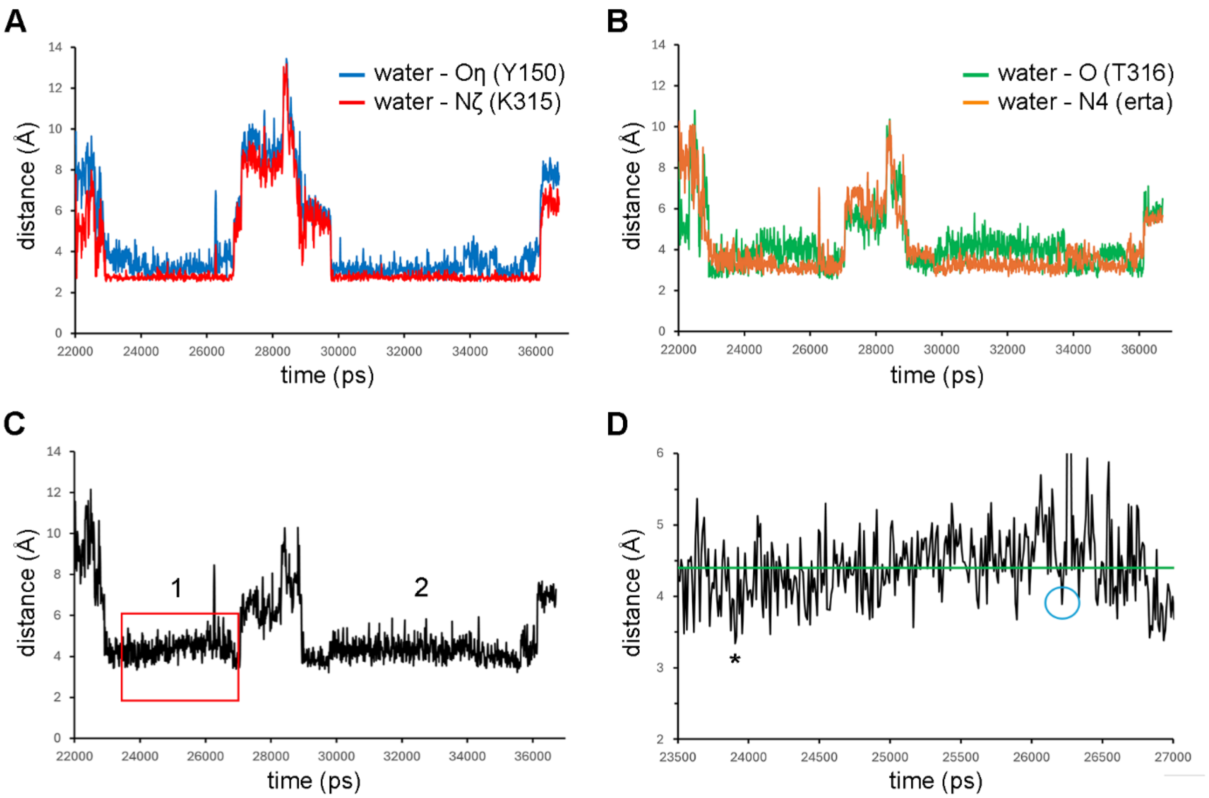

Figure S4. Analysis of the contacts with a representative water molecule in the DWP in the MD simulation of the ADC-1-ertaB complex. (A) Plot of the distances between a representative water molecule and the Oη atom of Tyr150 (blue trace) and the Nζ atom of Lys315 (red trace). This particular water enters the DWP twice and remains for a total of 10.3 ns. **(B)** Plot of the distances between the representative water molecule and the main chain O atom of Thr316 (green trace) and the N4 atom of ertapenem (orange trace). **(C)** Distance between the representative water and the C7 atom of ertapenem. The water molecule in question enters the pocket at around 23 ns (zone 1) and then moves to an adjacent site at 27 ns. It re-enters the pocket at 29 ns (zone 2) and finally leaves at 36 ns. **(D)** Expanded view of the red box shown in panel **C** between 23.5 and 27 ns. The green line is the average water-C7 distance of 4.4 Å. The representative frame at 26.22 ns used in Figure 4B is indicated by the cyan circle. The frame at 23.91 ns where the water comes to within 3.3 Å (also shown in Figure 4B) is indicated by the asterisk.

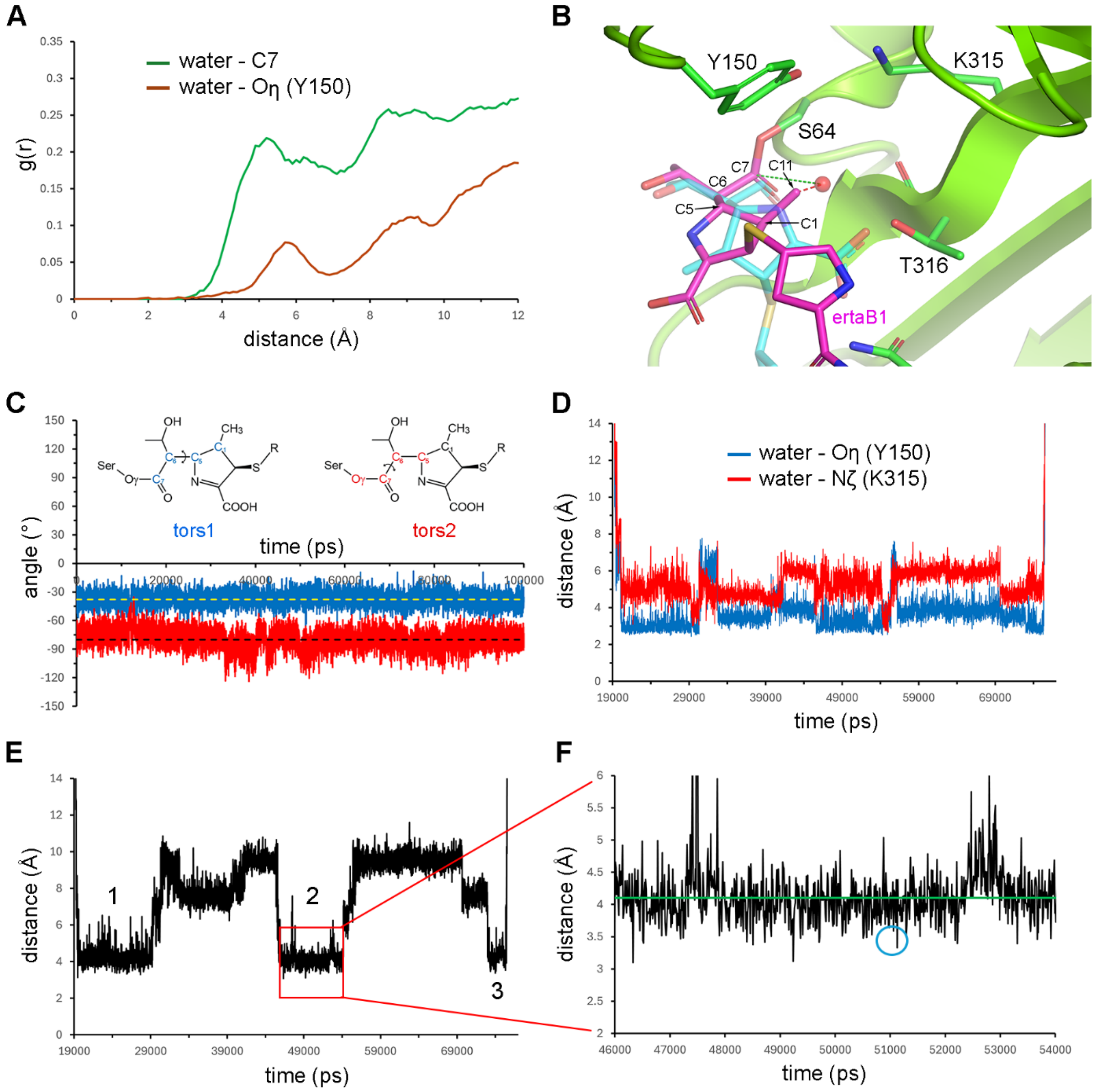

Figure S5. Analysis of the DWP in the MD simulations of the ADC-1$_{TM}$-ertaB1 and ADC-1$_{TM}$-ertaB2 complexes. (A) RDF showing the probability distribution of water molecules as a function of distance to the C7 atom (green trace) and the Oη atom of Tyr150 (brown trace) from the 100 ns MD simulation of the ADC-1$_{TM}$-ertaB1 model. The lack of water molecules closer than 5 Å to both atoms indicates no water molecules enter the DWP. **(B)** Representative frame from the ADC-1$_{TM}$-ertaB1 MD simulation. The C11 methyl of ertapenem (magenta sticks) projects into the DWP and would preclude any potential deacylating water molecules from entering. The approximate position for a viable deacylating water taken from the ADC-1-ertaB MD trajectory is represented by the red sphere (the green dashed line represents a distance of 3.3 Å). This water would be ~0.7 Å from the C11 atom (red dashed line). The conformation of ertapenem in ADC-1-ertaB is shown as semi-transparent cyan sticks. **(C)** Plot of two torsion angles, tors1 (blue trace)

and tors2 (red trace) from the ADC-1_{TM}-ertaB1 MD simulation. The diagrams above the plot specify the two angles that were monitored. Both plots are tightly clustered around their averages (the yellow and black dashed lines for tors1 and tors2, respectively), which shows that the ertapenem molecule remains in the same conformation throughout the simulation, with the C11 methyl group occupying the DWP. **(D)** Plot of the distances between a representative water molecule and the Oη atom of Tyr150 (blue trace) and the Nζ atom of Lys315 (red trace) from the MD simulation of ADC-1_{TM}-ertaB2. **(E)** Plot of the distance between the representative water and the C7 atom of ertapenem from the MD simulation of ADC-1_{TM}-ertaB2. The water molecule enters the pocket at ~19 ns (zone 1), moves to an adjacent site at 29 ns, and re-enters at ~46 ns (zone 2) for 8 ns. There is a third short residency in the pocket between 72.5 and 75.5 ns (zone 3) before the water finally leaves the active site. **(F)** Expanded view of the red box shown in panel **E** between 46 and 54 ns. The green line is the average water-C7 distance of 4.1 Å. The representative frame at 51.16 ns used in Figure 5D is indicated by the cyan circle.

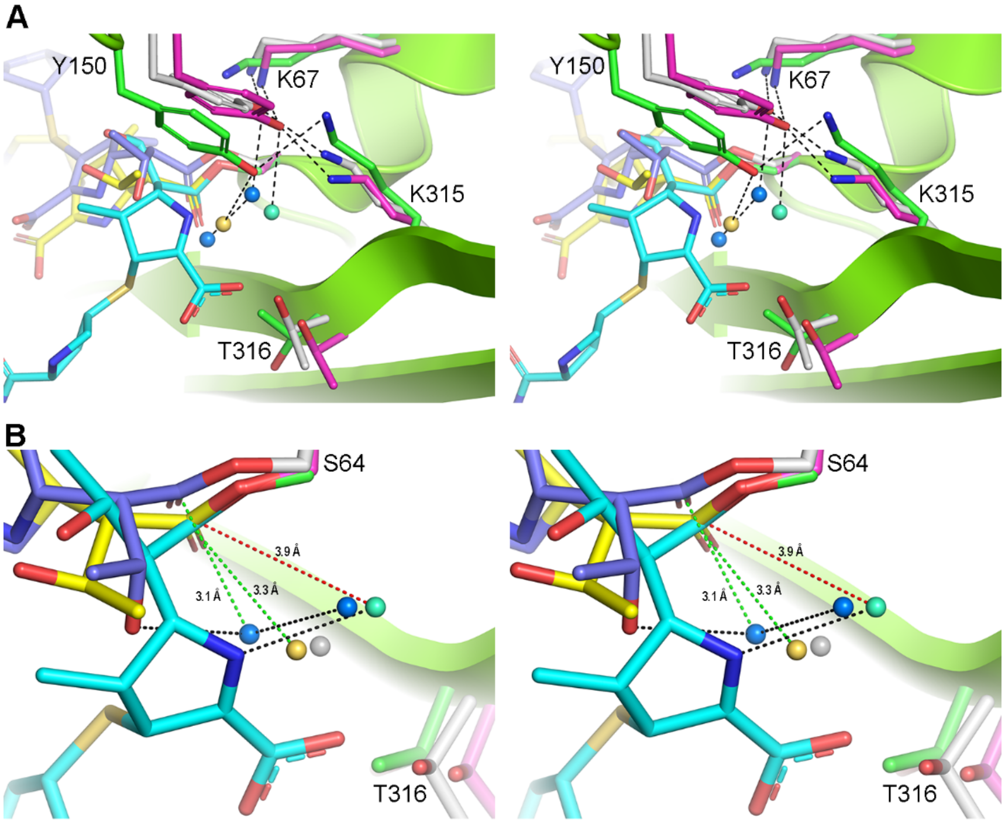

Figure S6. Superposition of the DWP from MD simulations. (A) Stereoview of the representative frames from the ADC-1-ertaB (magenta sticks and cyan ertapenem), ADC-1_{TM}-ertaA (gray sticks and light blue ertapenem), and ADC-1_{TM}-ertaB (green ribbons and sticks and yellow ertapenem) MD simulations used to generate Figures 4B, 5C, and 5D. The frames were superimposed based on the main chain atoms of six residues encompassing Ser64 and Lys67. The water molecules which reside in the DWP are shown, the light green sphere for ADC-1-ertaB, the two blue spheres for ADC-1_{TM}-ertaA, and the yellow sphere for ADC-1_{TM}-ertaB. **(B)** Close up of the superposition in panel A showing the same water molecules. The gray sphere represents the alternate water position from the ADC-1-ertaB frame shown in Figure 4B, where the pyrroline ring has reoriented and the water comes closer to the C7 atom. The green dashed lines indicate water-C7 distances that could lead to productive deacylation, and the red dashed line indicates that the water is too far from the C7 atom for hydrolysis. In both panels, hydrogen bonding interactions are shown as dashed black lines.

REFERENCES

1. Weiss MS. 2001. Global indicators of X-ray data quality. J Appl Crystallogr 34:130-135. doi:10.1107/S0021889800018227.

2. Karplus PA, Diederichs K. 2012. Linking crystallographic model and data quality. Science 336:1030-1033. doi:10.1126/science.1218231.

3. Chen VB, Arendall WB, 3rd, Headd JJ, Keedy DA, Immormino RM, Kapral GJ, Murray LW, Richardson JS, Richardson DC. 2010. MolProbity: all-atom structure validation for macromolecular crystallography. Acta Crystallogr Sect D: Biol Crystallogr 66:12-21. doi:10.1107/S0907444909042073.
